# Lipid Extraction from Various Species of Wet Microalgae Using Liquefied Ammonia

**DOI:** 10.1021/acsomega.5c00212

**Published:** 2025-04-18

**Authors:** Kiyoshi Sakuragi, Maromu Otaka

**Affiliations:** Energy Transformation Research Laboratory, Central Research Institute of Electric Power Industry, 2-6-1 Nagasaka, Yokosuka, Kanagawa 240-0196, Japan

## Abstract

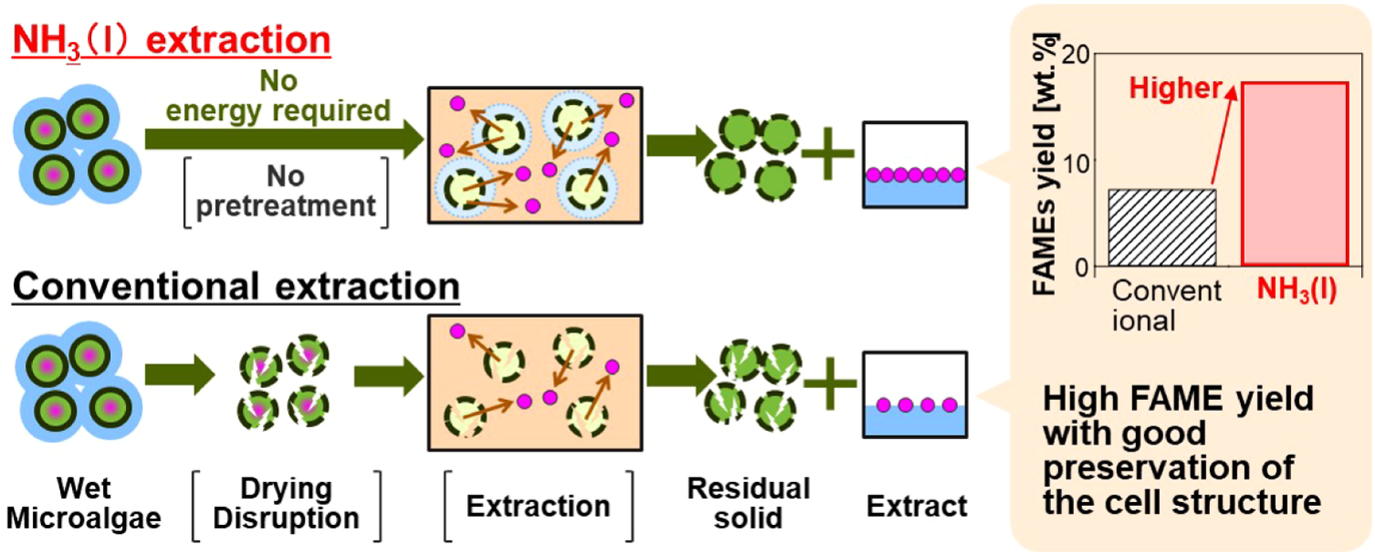

Energy-intensive drying or cell-disruption processes in lipid extraction procedures reduce efficiency and increase the cost involved in producing biofuels from microalgae. Recently, a simple method was proposed for extracting lipids directly from microalgae by utilizing liquefied ammonia (NH_3_(l)), which eliminates the energy-intensive processes. In the present study, the versatility of NH_3_(l) extraction was assessed by applying it to six microalgal species with different contents of lipids. This method directly separated 25.6–70.6 wt % of the dry weight of the sample as crude extract. The total fatty acid methyl ester yield was 5.5–17.4 wt %, comparable to or exceeding that obtained by traditional approaches. The crude extract was highly enriched with valuable C12–C24 fatty acids. Furthermore, scanning electron microscopy showed that after NH_3_(l) extraction, the cellular structure of the residue was preserved. These results suggest that employing the NH_3_(l) extraction method enhances applicability and consequently increases the process value by separating components into extract and useful residual solids.

## Introduction

Microalgae are a promising source of valuable components such as lipids, pigments, carbohydrates, and proteins.^1−3^ These constituents can be transformed into valuable products, including biofuels, materials, and cosmetics. Specifically, the lipid fractions in microalgae can be transformed into aviation fuel and biodiesel.^4,5^ Introducing algae-based biofuels could contribute to reducing CO_2_ emissions in the transportation sector, an area that often presents challenges in terms of decarbonization. This shift could contribute toward sustainable energy systems.^5−8^

A key technological challenge in microalgae utilization is the extraction of components from algal cells. Except for a few algal species that produce target components extracellularly, the majority require drying and cell-disruption processes to maximize solvent-based extraction efficiency.^7,9,10^ Conventional solvent extraction methods utilize either a single solvent or mixtures of organic solvents.^11,12^ In addition, several extraction methods involving ionic liquids, supercritical fluids, liquefied gas, and eutectic solvents, have been proposed to extract valuable components.^11−13^

Among the various solvents, hexane has been widely used for lipid extraction from bioresources at an industrial scale. However, several studies have identified the hexane-based extraction process as a major environmental hotspot in algal biofuel production.^14−17^ In particular, the energy required for moisture removal accounts for about 84% of the total energy consumption in microalgal biodiesel production,^14^ and the cell disruption step often demands more energy than the calorific value of the resulting biofuel.^15^ Lipid extraction has also been reported to contribute up to 94% of total greenhouse gas emissions across the entire process chain, which is attributed in part to the extensive use of chemicals such as hexane.^16^ In addition, the economic viability of reusing or converting the residual solid after extraction into valuable byproducts has been emphasized in algal biofuel production.^18^ These findings highlight the pressing need to develop more energy-efficient and environmentally sustainable alternatives to conventional hexane-based extraction methods.

A straightforward method has been proposed for extracting lipids from microalgae with high moisture content by utilizing liquefied ammonia (NH_3_(l)).^19^ Ammonia has the unique ability to dissolve various organic and inorganic compounds. Although ammonia production is currently an energy-intensive process, it does not emit CO_2_ during combustion and exhibits favorable storage and transportation properties. Consequently, with advancements in environmentally sustainable ammonia production technologies, a target of 3 million tons by 2030 and 30 million tons by 2050 has been set for its utilization as a hydrogen carrier in Japan.^20^ Furthermore, ammonia can be easily recovered as a solvent through gas–liquid phase transitions at near-ambient temperatures^21^ and ultimately incinerated with heat recovery,^22^ which makes it a potentially low-environmental-impact solvent choice. These properties make NH_3_ suitable as the extraction solvent for microalgal lipids.

This study applied the NH_3_(l) extraction process for lipid extraction from industrially relevant and fatty acid-producing microalgal species to assess the efficacy and broad applicability of this method. The crude extract yields, fatty acid methyl ester (FAME) profiles, and morphological changes in residual cell characteristics were compared with those obtained using conventional methods, and the potential of the NH_3_(l) method for producing valuable products was subsequently evaluated.

## Results and Discussion

### Microalgal Sample Lipid Extraction

**Figure 1:**
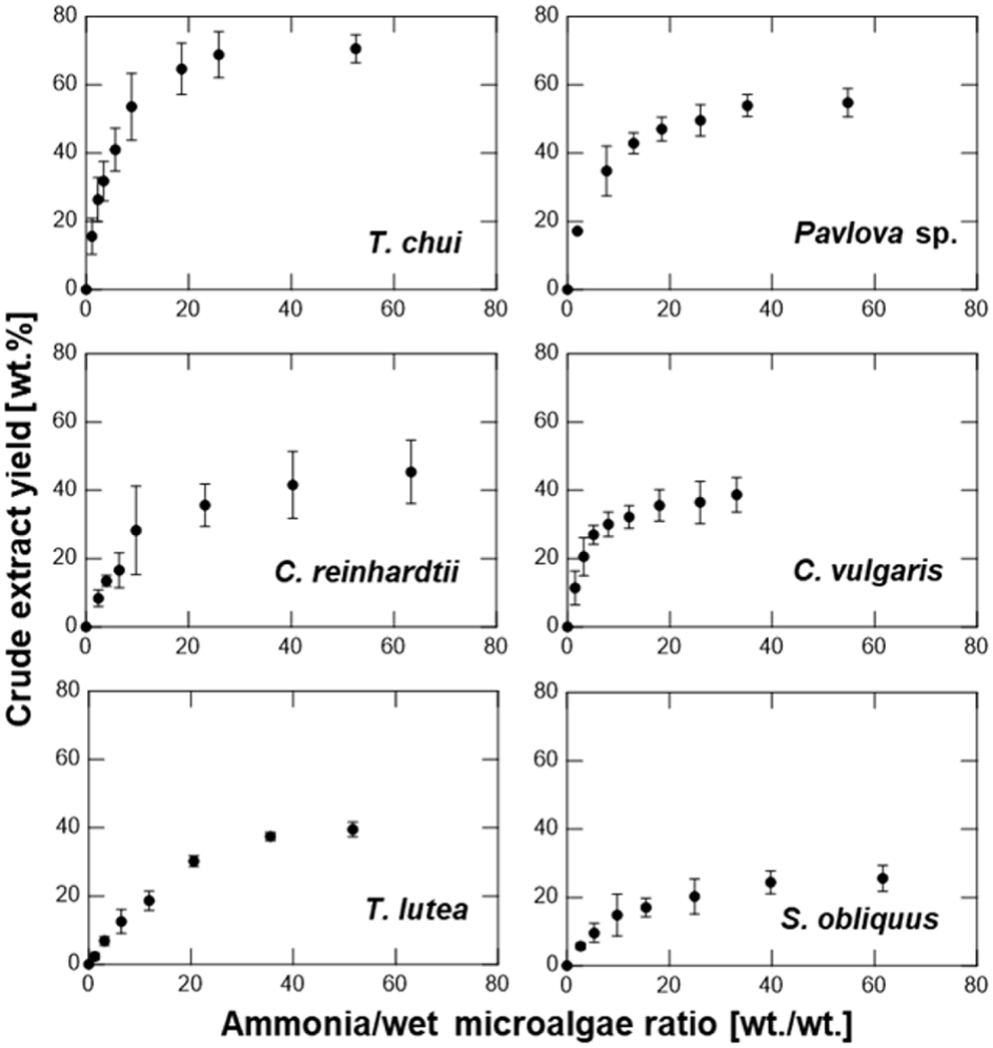
Crude extract yields from six microalgal species without drying and cell-disruption using liquefied NH_3_ extraction.

**Figure 2:**
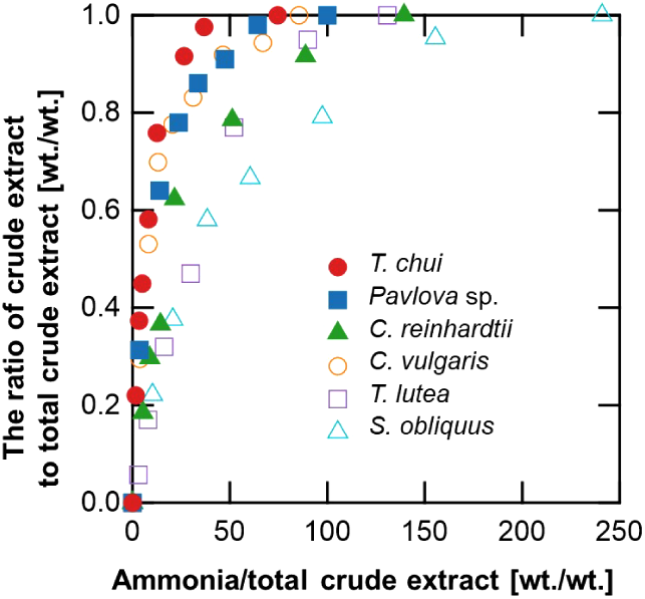
Correlation between the ratio of crude extract and total crude extract and the ratio of NH_3_ consumption and total crude extract. The total crude extract was presented when sufficient NH_3_(l) had passed through and the extraction had reached equilibrium. The ratio of crude extract to total crude extract (wt/wt) = (wt of the crude extract/wt of the total crude extract).

**Figure 3:**
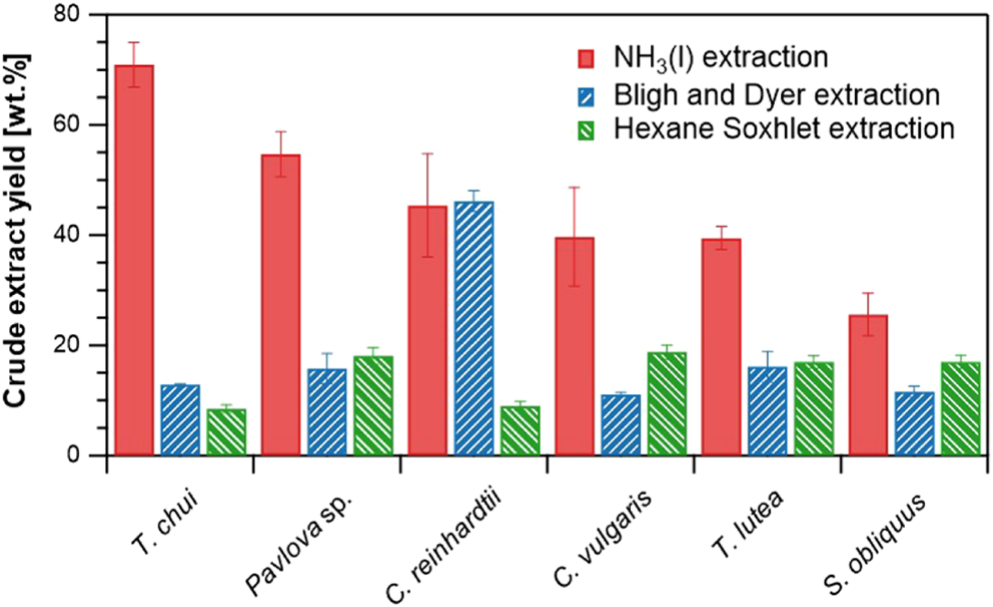
Crude extract yields from six microalgal species subjected to the three extraction methods.

Three different extraction methods were applied across six microalgal species to evaluate the lipid extraction efficiency: NH_3_(l), Bligh and Dyer (BD), and hexane Soxhlet (HS). The correlation between the weight of ammonia that permeated the microalgae and the crude extract yield is shown in Figure 1. All microalgal samples exhibited an increasing trend in crude extract yield with increasing weight of ammonia. The extraction process was largely complete for all microalgal species when the ammonia/wet microalgae weight ratio reached 40:1. Figure 2 shows the correlation between the weight ratio of ammonia consumption to the total crude extract and the weight ratio of crude extract to the total crude extract of each microalga. The crude extract is presented as a ratio of the total crude extract at equilibrium. Because the amount of crude extract contained in each microalgal sample was different, the weight of ammonia that passed through (Figure 2) is shown as a ratio to the total crude extract of the tested microalgae. The algal species exhibited varying ratios of the weight of ammonia required to achieve maximum crude extract, with the order from lowest to highest weight as follows: *Tetraselmis chui*, *Pavlova* sp., *Chlorella vulgaris*, *Chlamydomonas reinhardtii*, *Tisochrysis lutea*, and *Scenedesmus obliquus*. Figure 3 presents a comparative analysis of the crude extract yields achieved with the three different methods. The crude extract yield of the NH_3_(l) extraction method in the bar graph corresponds to the plateau value of the results shown in Figure 1, signifying the crude extract yield when sufficient NH_3_(l) had passed through and the extraction reached equilibrium. The crude extract yields achieved through the NH_3_(l) extraction method ranged from 25.6 to 70.6 wt % of dry-weight biomass, with species listed in decreasing order of yield: *T. chui*, *Pavlova* sp., *C. reinhardtii*, *C. vulgaris*, *T. lutea*, and *S. obliquus*. Species that required a lower weight ratio of ammonia for maximum crude extract yield tended to exhibit higher crude extract yields. The commonly used BD extraction method and the HS extraction method yielded 11.1–46.2 wt % and 8.5–18.8 wt % crude extracts, respectively. Although lipid content varies with cultivation conditions, cell disruption conditions, and microalgal species, the crude extract yields achieved through these conventional methods were comparable to those obtained in previous studies (Table 1).^7,23−27^ Therefore, these results demonstrated that the NH_3_(l) extraction method achieved a higher crude extract yield than the conventional extraction method. Moreover, these results suggest that the NH_3_(l) extraction method directly extracted crude extracts at ambient temperature from various high-moisture microalgal species without requiring cell disruption. Additionally, compared to the hexane extraction method widely used in industrial applications, the NH_3_(l) extraction method is expected to significantly reduce solvent utilization. The effectiveness of NH_3_(l) extraction varied among species, whereas the HS extraction method showed less variation in yields. Among the species, relatively high crude extract amounts were obtained through the NH_3_(l) extraction method for *T. chui* and the BD extraction method for *C. reinhardtii*. However, these methods resulted in lower yields for *S. obliquus*. Conversely, the HS extraction method resulted in lower yields from *T. chui* and *C. reinhardtii* but higher yields from *S. obliquus*. These variations may partly result from the robust cell wall structure of *S. obliquus*, consisting of rigid layers including an inner cellulose layer and an outer trilaminar algaenan layer, which makes cell disruption challenging.^28,29^

**Table 1:** Comparison of Crude Extract Yields with Reported Lipid Contents

	Crude extract yield [wt %]			
Microalgae species	NH_3_(l)	Bligh and Dyer	Hexane Soxhlet	Reported Lipid content [wt %]^7,23−27^
*T. chui*	70.9 ± 4.1	12.9 ± 0.1	8.5 ± 0.7	12.6–23.5
*Pavlova* sp.	54.7 ± 4.1	15.8 ± 2.7	18.1 ± 1.5	30.9–35.5
*C. reinhardtii*	45.4 ± 9.4	46.2 ± 1.9	9.0 ± 0.8	7.6–34.5
*C. vulgaris*	39.7 ± 8.9	11.1 ± 0.4	18.8 ± 1.3	5.0–58.0
*T. lutea*	39.5 ± 2.1	16.1 ± 2.8	17.0 ± 1.1	6.5–33.0
*S. obliquus*	25.6 ± 3.9	11.6 ± 1.0	17.0 ± 1.2	2.4–55.0

### Scanning Electron Microscopy Analysis of Control Samples and NH_3_(l) Extracted Residue

**Figure 4:**
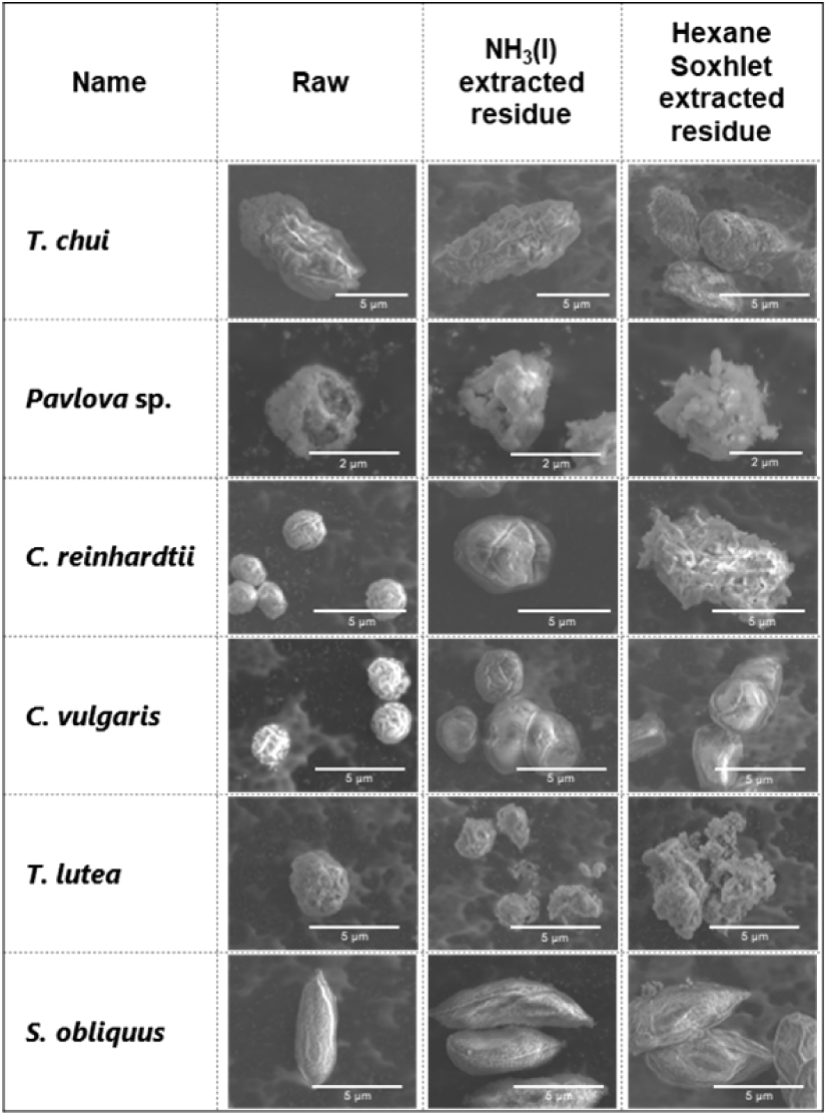
Scanning electron microscopy (SEM) of microalgal samples before and after extraction. A slight expansion in cell size was observed in the NH_3_(l) extraction residues of *Chlamydomonas reinhardtii* and *Chlorella vulgaris*. Cell morphology was better preserved in all tested algal species’ NH_3_(l) extraction residues compared to the hexane Soxhlet extraction residues.

Scanning electron microscopy (SEM) images of the original raw samples, NH_3_(l) extraction residue, and HS extraction residue are shown in Figure 4. Because the BD extraction method produces multiple solids in the intermediate and sedimentation layers during the extraction process, the residues are not compared here. Although some flagella were damaged, the original raw samples exhibited spherical or elliptical cellular structures, indicating intact cell wall structures. Notable deformation, accompanied by changes in the cell wall structure, was observed in the HS extraction residues for most algal species. This observation is attributed to the drying and homogenizing cell disruption treatment preceding the HS extraction. *S. obliquus* displayed minor cellular structural changes, which may explain the low crude extract yield. In contrast, the NH_3_(l) extraction residues showed slight expansion in cell size and partial deformation in several species, but the cell morphology was better preserved than in the HS extraction residues. Previous studies have shown that alkaline pretreatment^30^ and rapid changes in osmotic pressure inside and outside the cell^24,31^ can cause changes in the cell structure. Moreover, studies on plant biomass have reported expansion of fiber structure containing cellulose crystals^32^ and ester bond cleavage^33^ following NH_3_(l) treatment. Similar changes may occur in microalgae subjected to NH_3_(l) extraction; however, high crude extract yields could be obtained with good cell structure preservation. The NH_3_(l) extraction method, which preserves the cell structure while achieving high lipid yields, may unlock new high-value applications, such as material utilization of residues, which is challenging with conventional extraction methods relying on cell disruption.

### Fatty Acid Composition

**Figure 5:**
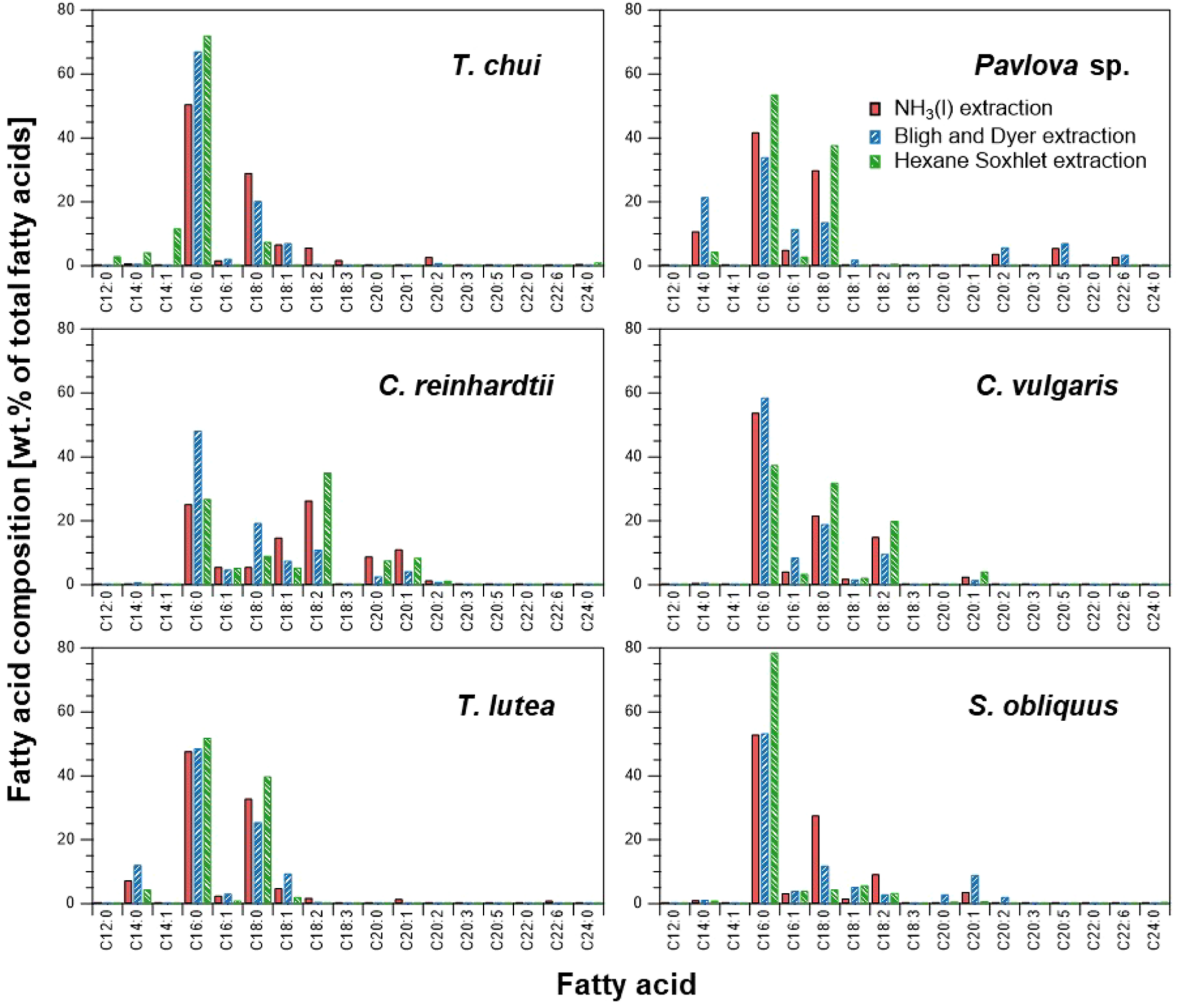
Fatty acid profiles of extracts obtained through NH_3_(l), Bligh and Dyer, and hexane Soxhlet extraction methods.

The fatty acids in the crude extracts derived through the NH_3_(l), BD, and HS extraction methods were transformed into FAMEs and analyzed to determine their composition (Figure 5). The fatty acid profiles varied depending on the extraction method and microalgal species. However, the fatty acid profiles obtained through the NH_3_(l) extraction method were generally consistent with those extracted using conventional methods. These extracts contained valuable C12–C24 fatty acids, including palmitic acid (C_16:0_), palmitoleic acid (C_16:1_), stearic acid (C_18:0_), oleic acid (C_18:1_), linoleic acid (C_18:2_), and eicosapentaenoic acid (EPA, C_20:5_).^34^ When comparing the FAME composition ratio in the extracts, the NH_3_(l) and BD extraction methods yielded long-chain unsaturated fatty acids comprising 20 or more carbon atoms, such as EPA. These fatty acids included high value fatty acids, suggesting their potential for high-value applications.

**Figure 6:**
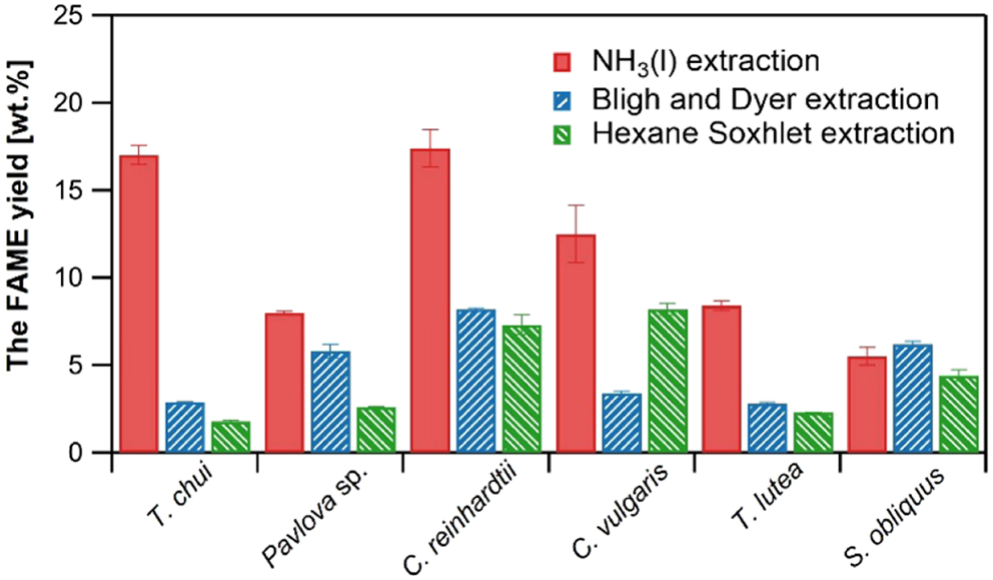
Fatty acid methyl ester (FAME) composition of extracts from the NH_3_(l), Bligh and Dyer, and hexane Soxhlet extraction methods.

Figure 6 shows the FAME yields determined from the dry weight of the microalgae extracted using the NH_3_(l), BD, and HS extraction methods. The FAME yields varied across extraction methods and the algal samples. The FAME yield achieved using the NH_3_(l) extraction method was 5.5–17.4 wt %. The FAME yields from the NH_3_(l) extraction method consistently exceeded those obtained by the BD (2.8–8.2 wt %) and HS extraction methods (1.8–8.2 wt %), except for *S. obliquus*. Despite the pure lipids generally having lower solubility in polar solvents like NH_3_(l), the FAME yield in NH_3_(l) exceeded that obtained with the nonpolar solvent hexane. As previously suggested,^35^ solubility alone does not determine the efficiency of lipid extraction from wet microalgal biomass. For example, when employing polar solvents such as 96 vol % ethanol and 75 vol % 2-BuOH, the influence of cell disruption has minimal effect on lipid yield from wet microalgae due to the solvents’ high permeability.^36^ Consequently, while a higher cell disruption typically enhances lipid extraction efficiency, its effect depends on the type of solvent used. In the present study, the high permeability of NH_3_(l) likely contributed to the high FAME yield obtained from nondisrupted wet microalgae. The FAME yields to the crude extract yield ratios were 0.2–0.4 for the NH_3_(l), 0.2–0.6 for the BD, and 0.1–0.8 for the HS methods. Because crude extracts from the NH_3_(l) extraction method contain significant amounts of nonfatty acid components, they may require refinement depending on their intended use.^37,38^ Traditional refining processes typically consist of multiple steps, including degumming, neutralization, bleaching, winterization or fractionation, and deodorization. In comparison with the HS extraction method, crude extracts derived through the NH_3_(l) extraction method from *T. chui*, *T. lutea*, and *Pavlova* sp., which yielded particularly high FAMEs, exhibited higher levels of long-chain unsaturated fatty acids. Nevertheless, the NH_3_(l) extraction FAME yields of C16 and C18 fatty acids, which are considered suitable for biofuels, were comparable to or exceeded those achieved by HS extraction (Figure S1).

The experimental results suggest that the NH_3_(l) extraction method is highly effective for extracting lipids from various microalgae. However, compared to the well-established HS extraction method, the assessment of the influence of extraction conditions and microalgae components on the extraction characteristics remains insufficient. For large-scale applications, challenges such as ammonia handling safety, energy-efficient operation, and cost-effectiveness need to be addressed. Such an assessment is crucial for producing high-quality biofuels and enhancing value. Future research should focus on acquiring data regarding optimal extraction conditions and developing scalable process designs essential for industrial applications.

## Conclusions

The NH_3_(l) extraction method efficiently yielded substantial amounts of crude extract, which was rich in long-chain unsaturated fatty acids, from six microalgae species at ambient temperature. Furthermore, the crude extracts from this method yielded sufficient FAMEs when compared to the conventional methods. Additionally, the extraction residues from the NH_3_(l) extraction method retained their cell structures, indicating that the cell disruption pretreatment was not necessary for lipid extraction from these microalgae. The NH_3_(l) lipid extraction method is effective and shows promise for application in biofuel production as well as the new value-added uses of preserved cell structures.

## Methods

### Microalgal Sample Lipid Extraction

*Chlamydomonas reinhardtii* cc1010 and *S. obliquus* UTEX-393 with moisture contents of 87.2 and 70.2 wt %, respectively, were kindly provided by the Institute of Microalgal Technology (Hiroshima, Japan). *Chlorella vulgaris* CK-5 with a moisture content of 82.4 wt % was purchased from Chlorella Industry Co. Ltd. (Tokyo, Japan). *Tisochrysis lutea* CCMP1324, *Pavlova* sp. (strain: Pavlovophyceae) CCPM459, and *T. chui* PLY429 were purchased from Reed Mariculture Inc., Campbell, CA, USA, and contained 73.0, 74.5, and 81.6 wt % moisture, respectively. The microalgal samples were stored at −20 °C until further analysis. The moisture content of wet samples was calculated as the weight lost when dried at 40 °C in a vacuum oven.

The NH_3_(l) extraction setup and procedure were implemented as previously described.^19^ A wet sample (1.0 g) was placed in a pressure-resistant vessel and extracted with NH_3_(l) at 20 °C and 0.85 MPa, using an approximate flow rate of 10 cm^3^/min. To compare the extraction characteristics across different extraction methods, the BD and HS extraction methods were conducted according to a previous report.^19^ The extraction yield was determined using the weight of the crude extract obtained after distillation, as expressed by the following equation:
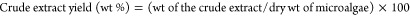


### FAME Composition of the Extracts

The fatty acid composition of the extracts was determined after methylation using a methylation kit (Nacalai Tesque Inc., Kyoto, Japan). A gas chromatograph–mass spectrometer was used to analyze the FAMEs as previously described.^19^ The FAME concentrations of the sample were measured by correlating their peak areas with those obtained from tridecanoic acid methyl ester, which served as the internal standard, the NIST11 library, and a standard FAME solution (Supelco 37 Component FAME Mix, Sigma-Aldrich Co. LLC, St. Louis, MO, USA).

### SEM of Unextracted Samples and Extracted Residue

Initially, the samples were treated with 2.0% (v/v) glutaraldehyde solution and incubated at 4 °C for 3 h, followed by three rinses with 0.1 mol/dm^3^ phosphate buffer solution (PBS, pH 7.4). Microalgal cells were then fixed with a 1% (v/v) osmium tetroxide solution at 4 °C for 1 h, followed by three additional rinses with PBS. The washed samples were then dehydrated using a series of ethanol solutions (30, 50, 70, 90, 95, and 100%). Finally, the samples were fixed in *tert*-butyl alcohol, freeze-dried, and mounted on carbon paper for the SEM observation (S-4800, Hitachi High-Tech Corp., Tokyo, Japan).

## Data Availability

All data generated or analyzed during this study are included in this published article and its Supporting Information files.

## Supporting Information Available

Figure S1: graphic representation of estimated fatty acid methyl ester (FAME) yields from microalgae obtained using the NH_3_(l), Bligh and Dyer, and hexane Soxhlet extraction method (PDF)
