# Supplementary material for: Lipid Extraction from Various Species of Wet Microalgae Using Liquefied Ammonia
Source: ACS Omega. 2025 Apr 18;10(16):16642–7. doi: 10.1021/acsomega.5c00212 (PMC12044556; doi:10.1021/acsomega.5c00212)
Supplement: Supplementary file 1 — ao5c00212_si_001.pdf [file ao5c00212_si_001.pdf]

Supporting Information

Lipid extraction from various species of wet microalgae using liquefied ammonia

Kiyoshi Sakuragi[a,*] and Maromu Otaka[a]

[a]Energy Transformation Research Laboratory, Central Research Institute of Electric Power Industry, 2-6-1 Nagasaka, Yokosuka, Kanagawa 240-0196, Japan

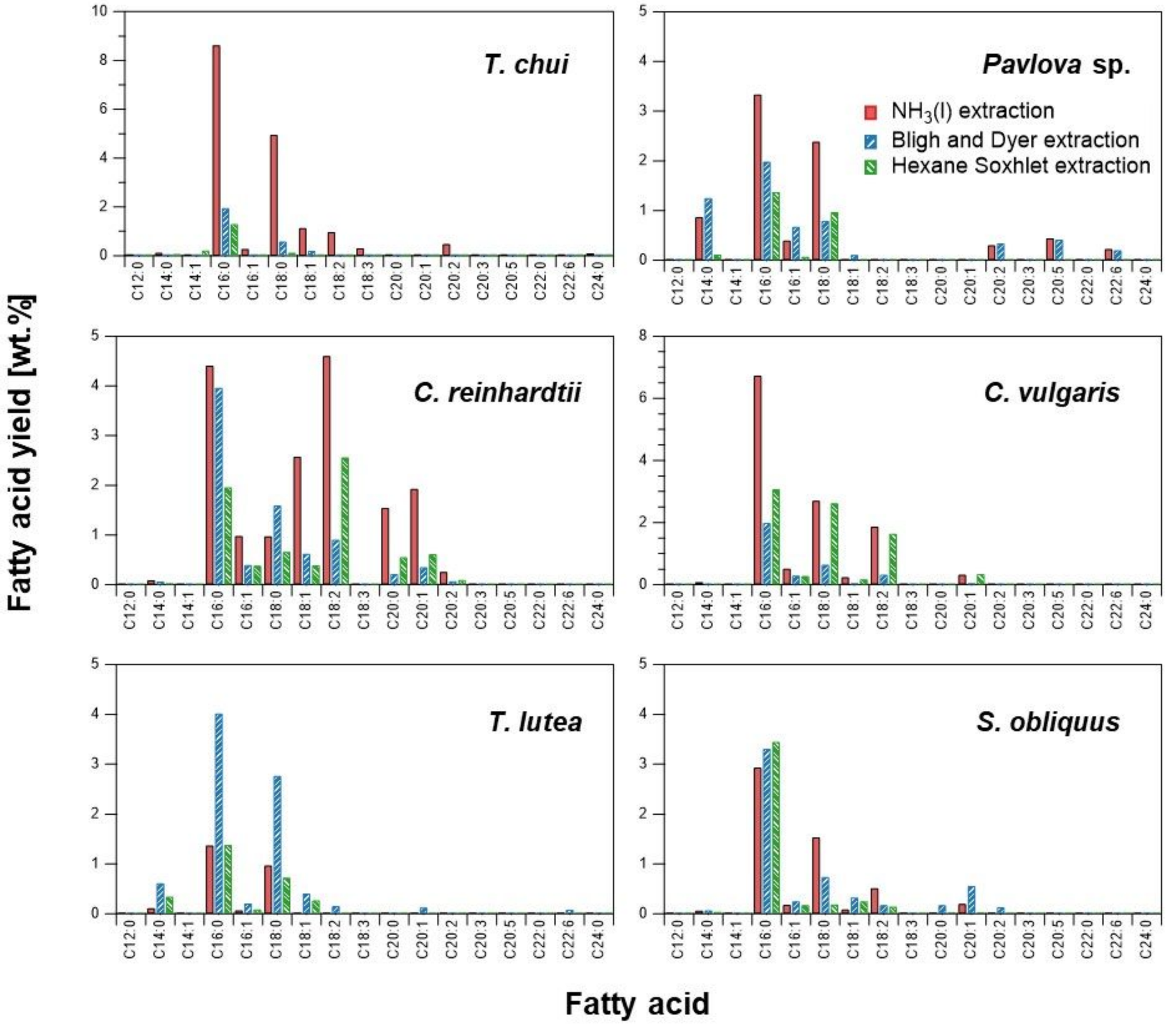

Figure S1. Fatty acid methyl ester (FAME) yields from microalgae obtained using the NH_3(l), Bligh and Dyer, and hexane Soxhlet extraction method.

The yields were calculated based on the area corresponding to the internal standard, as follows:

$$\text{Fatty acid yield (wt.\%)} = A / A_{EI} \times (C_{EI} \times V_{EI}) / m \times 100$$

Here, A represents the peak area of each fatty acid methyl ester (FAME), A_{EI} is the peak area corresponding to the internal standard, tridecanoic acid, C_{EI} is the concentration of the

tridecanoic acid solution (mg/mL), V_{EI} is the volume of the tridecanoic acid solution used (mL), and m is the mass of the sample injected (mg).
